# Metabolism of Diterpenoids Derived from the Bark of *Cinnamomum cassia* in Human Liver Microsomes

**DOI:** 10.3390/pharmaceutics13081316

**Published:** 2021-08-23

**Authors:** Su Min Choi, Van Cong Pham, Sangkyu Lee, Jeong Ah Kim

**Affiliations:** 1 BK21 FOUR Community-Based Intelligent Novel Drug Discovery Education Unit, College of Pharmacy, Kyungpook National University, Daegu 41566, Korea; ys01216@naver.com; 2 College of Pharmacy, Kyungpook National University, Daegu 41566, Korea; phamcong1990@gmail.com; 3 Research Institute of Pharmaceutical Sciences, Kyungpook National University, Daegu 41566, Korea; 4 Vessel-Organ Interaction Research Center, VOICE (MRC), College of Pharmacy, Kyungpook National University, Daegu 41566, Korea

**Keywords:** diterpenoids, anhydrocinnzeylanine, CYPs, human liver microsomes, metabolism

## Abstract

*Cinnamomum cassia* L. is used as a spice and flavoring agent as well as a traditional medicine worldwide. Diterpenoids, a class of compounds present in *C.* *cassia*, have various pharmacological effects, such as anti-inflammatory, antitumor, and antibacterial activities; however, there are insufficient studies on the metabolism of diterpenoids. In this study, the metabolism of seven diterpenoids, namely, anhydrocinnzeylanol, anhydrocinnzeylanine (AHC), cinncassiol A, cinncassiol B, cinnzeylanol, cinnzeylanone, and cinnzeylanine, obtained from the bark of *C. cassia* was studied in human liver microsomes (HLMs). All studied diterpenoids, except for AHC, exhibited strong metabolic stability; however, AHC was rapidly metabolized to 3% in HLMs in the presence of β-NADPH. Using a high-resolution quadrupole-orbitrap mass spectrometer, 20 metabolites were identified as dehydrogenated metabolites (**M1**–**M3**), dehydrogenated and oxidated metabolites (**M4**–**M10**), mono-oxidated metabolites (**M11**–**M13**), or dioxidated metabolites (**M14**–**M20**). In addition, CYP isoforms involved in AHC metabolism were determined by profiling metabolites produced after incubation in 11 recombinant cDNA-expressed CYP isoforms. Thus, the diterpenoid compound AHC was identified in a metabolic pathway involving CYP3A4 in HLMs.

## 1. Introduction

**Figure 1:**
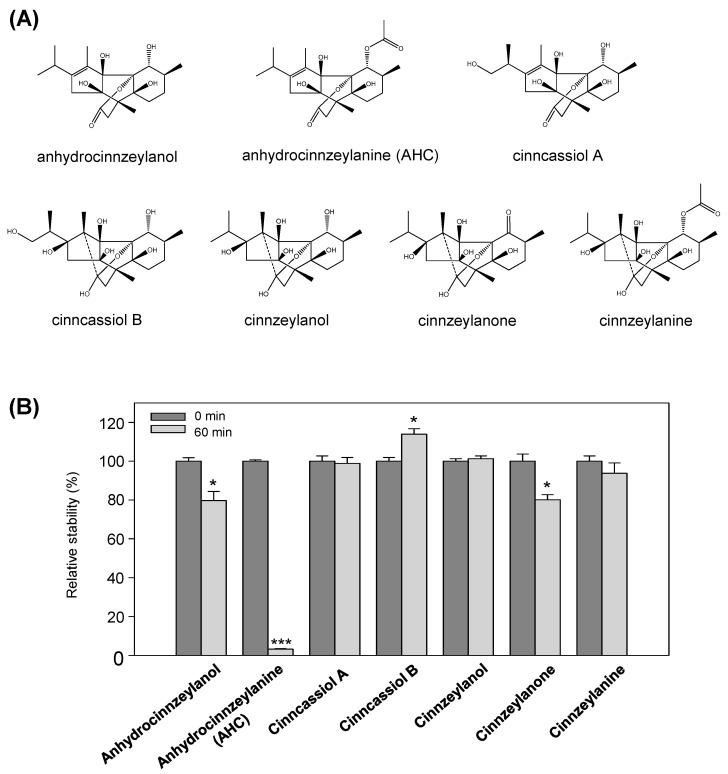
Determination of the metabolic stability of the seven diterpenoids. (**A**) Chemical structures of anhydrocinnzeylanol, cinncassiol A, anhydrocinnzeylanine (AHC), cinncassiol B, cinnzeylanol, cinnzeylanone, and cinnzeylanine. (**B**) Metabolic stability of the seven diterpenoids (10 µM) incubated with 0.5 mg/mL human liver microsomes in the presence of a β-NADPH-regenerating system at 37 °C for 0 or 60 min. Bars indicate standard error (*n* = 3). * *p* < 0.05 and *** *p* < 0.001.

*Cinnamomum cassia* L., which belongs to the family Lauraceae, is used as a spice and flavoring agent as well as a traditional medicine worldwide [1,2]. In traditional medicine, different parts of *C. cassia* are used for different therapeutic purposes [3]. For example, the leaves are used to treat headaches, chills, and abdominal pain, and the bark is used to treat tussis, gastrointestinal neurosis, diarrhea, edema, and cardiac palpitations. The constituents of the treatments are well known, along with the various medicinal effects of phenylpropanoids, sesquiterpenoids, lignans, and diterpenoids isolated from *Cinnamomum* species, as shown in previous phytochemical studies [4,5]. Among these, diterpene compounds have been mainly identified in the leaves of *C. cassia* and were recently isolated from the bark [6,7]. Anhydrocinnzeylanol, anhydrocinnzeylanine (AHC), cinncassiol A, cinncassiol B, cinnzeylanol, cinnzeylanone, and cinnzeylanine have been extracted from the bark of *C. cassia* and classified as diterpenoids [2,8,9,10,11] (Figure 1). The pharmacological effects of diterpenoids isolated from *C. cassia* bark include anti-inflammatory, antitumor, and antibacterial activities; thus, these compounds have attracted attention as a new natural active class [12,13,14].

Although the activities of diterpenes derived from *C. cassia* have been reported, research results have not been reported on the metabolism of these compounds. In the initial stages of novel drug development, the study of the metabolism of drug candidates is closely linked to the success of the process. Therefore, for natural active substances to be considered valuable drugs, it is necessary to elucidate their metabolic processes. Notably, metabolism is a process that increases the water solubility of absorbed xenobiotics and converts them into forms that can be easily excreted from the body [15,16]. However, the process of drug metabolism sometimes remains incomplete, leading to increased toxicity of bioactivation [17].

A liquid chromatography–mass spectrometry (LC–MS) system capable of high-resolution (HR) analysis is used as a common platform for drug metabolism studies. In particular, HR-MS can confirm the exact elemental compositions of product ions with HR (>10,000 at full width at half maximum) and accurate mass (<5 ppm deviation) capabilities [18]. Therefore, HR-MS enables the accurate identification of metabolite structures by acquiring product ion information generated by the MS^2^ of target compounds. In addition to its use in the qualitative analysis of metabolites, LC–MS is widely used as an essential instrument for metabolic stability evaluation and pharmacokinetic research in the development of new drug candidates [19].

Therefore, metabolism studies of active substances can provide important data for predicting pharmacokinetics and toxicity evaluation. In this study, to examine the pharmacological properties of diterpenes isolated from *C. cassia*, we evaluated the metabolic stability of seven diterpenes and identified the metabolic pathway of AHC, which had the highest metabolic rate in human liver microsomes (HLMs). Seven diterpenes isolated from *C cassia* were selected based on their structural similarity. The structures of the generated metabolites of AHC were identified with high resolution and accuracy using a mass spectrometer, and the human CYP isoforms involved in the metabolism of AHC were identified; these data can be used to predict additional drug interactions.

## 2. Materials and Methods

### 2.1. Chemicals and Reagents

AHC, anhydrocinnzeylanol, cinncassiol A, cinncassiol B, cinnzeylanol, cinnzeylanone, and cinnzeylanine were isolated from *C. cassia* [11]. The purity of the compounds isolated from *C. cassia* for the metabolism study was >95.0%, as determined using high-performance liquid chromatography (HPLC) and NMR. Pooled HLMs (UltraPool HLM 50) and human recombinant cDNA-expressed recombinant CYP isoforms (CYP1A1, 1A2, 2A6, 2B6, 2C8, 2C9, 2C19, 2D6, 2E1, 3A4, and 3A5) were acquired from Corning Gentest (Woburn, MA, USA). A reduced nicotinamide adenine dinucleotide phosphate (β-NADPH)-generating system (NGS) was purchased from Promega (Madison, WI, USA). Tolbutamide (internal standard), formic acid, proadifen hydrochloride (SKF-525A), dipotassium hydrogen phosphate, and potassium dihydrogen phosphate were purchased from Sigma-Aldrich (St. Louis, MO, USA). MS-grade acetonitrile (ACN), methanol (MeOH), and water were obtained from Fisher Scientific (Pittsburgh, PA, USA). All other chemicals were of analytical grade and were used as received.

### 2.2. Metabolic Stability Studies

For metabolism profiling, 10 µM of each of the seven compounds isolated from *C. cassia* bark was added to 0.5 mg/mL of pooled HLMs and 0.1 M potassium phosphate buffer (pH 7.4). The HLMs were then preincubated at 37 °C for 1 min. After adding NGS, the reaction mixture was incubated at 37 °C for 60 min. After the 60 min incubation period, 50 µL of the reaction mixture was transferred to a new tube, and 50 µL of 100% ACN containing 0.1% formic acid and tolbutamide (internal standard) was added to terminate the reaction. The mixture was then vortexed, followed by centrifugation at 15,700× *g* (13,000 rpm) for 10 min (Eppendorf AG, Hamburg, Germany). Finally, 90 µL of the supernatant was transferred to a vial and injected into a C18 column for LC–MS/MS analysis.

### 2.3. Identification of AHC Metabolites in HLMs

To identify AHC metabolites, 10 µM AHC was incubated with 1 mg/mL pooled HLMs in 0.1 M potassium phosphate buffer (pH 7.4) at 37 °C for 60 min. The reactions were initiated by adding NGS to obtain a final incubation volume of 200 µL. All experiments were conducted in triplicate. The reaction was terminated by adding 400 µL of 100% ACN, and the mixture was vortexed and then centrifuged for 10 min at 15,700× *g*. Next, the supernatant (550 µL) was transferred to a new tube and dried using a speed-vac concentrator with a cold trap (Labconco, Kansas, MO, USA). Subsequently, the dried samples were reconstituted in 100 µL of 20% MeOH containing 0.1% formic acid before centrifugation at 15,700× *g* for 10 min. Finally, the mixture was injected into a liquid chromatograph with a tandem mass spectrometer for analysis.

### 2.4. Metabolism of AHC by SKF-525A: A Nonselective Inhibitor

To investigate CYP-mediated metabolism, 10 µM AHC was mixed with 1 mg/mL pooled HLMs and 0.1 M potassium phosphate buffer (pH 7.4). Then, SKF-525A, a general inhibitor of CYPs, was added at concentrations of 0.5, 1, and 5 mM. The reactions were initiated with the addition of NGS at 3 °C for 60 min. After 60 min, the reactions were terminated by adding 400 µL of 100% acetonitrile. The mixtures were then centrifuged at 15,700× *g* for 10 min. Subsequently, 550 µL of supernatant was dried in a vacuum concentrator. The residue was dissolved in 100 µL of 20% MeOH (0.1% formic acid), and 5 µL of this sample was injected into a C18 column for LC–MS/MS analysis.

### 2.5. Determination of Recombinant cDNA-Expressed CYP Isoforms Included in AHC Metabolism

To identify the metabolic enzymes for AHC, 10 µM AHC was incubated with 10 pmol of human recombinant cDNA-expressed CYP isoforms (CYP1A1, 1A2, 2A6, 2B6, 2C8, 2C9, 2C19, 2D6, 2E1, 3A4, and 3A5) in the presence of 0.1 M potassium phosphate buffer (pH 7.4). The reaction was initiated by adding 100 µL of NGS at 37 °C for 60 min. Then, the mixture was suspended by adding 400 µL of ice-cold 100% ACN. Following centrifugation at 15,700× *g* for 10 min at 4 °C, the supernatant was transferred to a new tube and evaporated using a speed-vacuum concentrator. Next, the dried sample was diluted with 100 µL of 20% MeOH containing 0.1% formic acid, and a 5 µL aliquot was injected into a C18 column for LC–MS/MS analysis.

### 2.6. LC–MS/MS

The metabolism screening of the seven compounds was performed using an HPLC system (Thermo Fisher Scientific, Bremen, Germany) equipped with an HPG-3200SD standard binary pump, a WPS-3000 TRS analytical autosampler, and a TCC-3000 SD column compartment. The HPLC system was coupled with an HR-MS (Q-Exactive Focus Quadrupole-Orbitrap MS; Thermo Fisher Scientific, Waltham, MA, USA) at the Mass Spectrometry Convergence Research Center. The full MS resolution and scan ranges were 70,000 and 60–900 *m/z*, respectively. The MS/MS resolution was 35,000. The mass spectra were obtained in negative ion electrospray mode. A heated electrospray ionization (HESI-II) probe was used as an ion generator, with nitrogen as the sheath gas at 35 aux units and auxiliary gas at 12 aux units. The mass spectrometer was operated in negative ionization mode. The other parameters were as follows: spray voltage, 3.5 kV; capillary temperature, 320 °C; S-lens radio frequency level, 50; and aux gas heater temperature, 400 °C.

The mobile phase consisted of 100% water with 0.1% formic acid (mobile phase A) and 100% ACN with 0.1% formic acid (mobile phase B). For metabolic stability, the gradient conditions were set as follows: 5% of B at 0.5–2.0 min, 5–50% of B at 2.0–4.5 min, 50% of B at 4.5–7.0 min, and 50–5% of B at 7.0–10.0 min at 40 °C with a flow rate of 0.2 mL/min. For metabolite identification, the gradient conditions were as follows: 10% of B at 0–5 min, 10–55% of B at 5–15 min, 55% of B at 15–23 min, and 55–10% of B at 23–30 min with a flow rate of 0.25 mL/min. Chromatographic separation was performed using a Kinetex C18 column (150 × 2.1 mm, 2.6 μm, XB-C18 100 Å, S/No: H19-070128; Phenomenex, Torrance, CA, USA). Data were analyzed using Xcalibur version 4.0 software (Thermo Fisher Scientific, Waltham, MA, USA).

### 2.7. Statistical Analysis

All data were expressed as the mean ± standard deviation. Statistical analysis was performed using IBM SPSS Statistics version 21 to determine significant differences. Values of *p* < 0.05 were considered statistically significant and are indicated with asterisks in the figures (i.e., * *p* < 0.05, ** *p* < 0.01, and *** *p* < 0.001).

## 3. Results

### 3.1. Metabolic Stability of the Seven Diterpenoids in HLMs

To confirm their metabolic stability, seven diterpenoids (AHC, anhydrocinnzeylanol, cinncassiol A, cinncassiol B, cinnzeylanol, cinnzeylanone, and cinnzeylanine) derived from the bark of *C. cassia* were incubated with HLMs in the presence of the NGS for 60 min (Figure 1B). The metabolic stability of each compound was determined according to the ratio of the peak area at 60 min to that at 0 min. This analysis showed that AHC showed the most rapid metabolization among the seven compounds. In contrast, the stabilities of anhydrocinnzeylanol and cinneylanone decreased by approximately 20%, whereas the remaining four compounds did not significantly decrease; thus, the six compounds other than AHC were metabolically stable in HLMs. Therefore, we identified the metabolites of AHC produced in HLMs.

### 3.2. Identification of Phase I Metabolites of AHC

**Figure 2:**
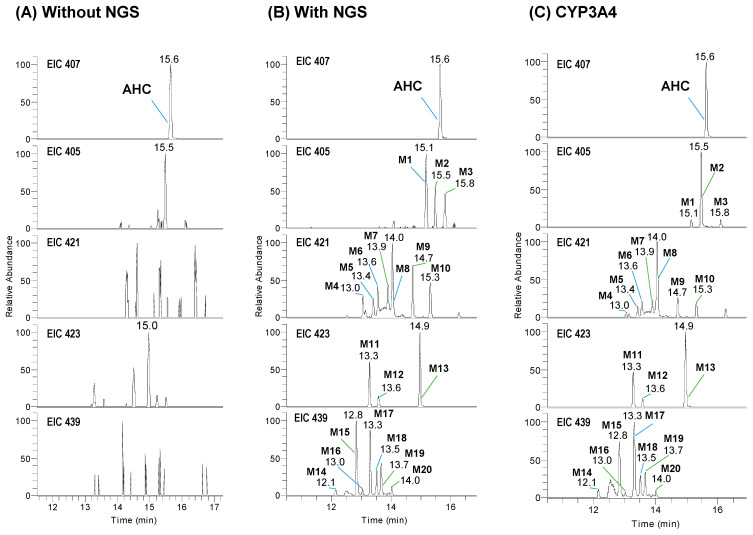
Extract ion chromatograms (EIC) for anhydrocinnzeylanine (AHC) and its metabolites. AHC (10 µM) was incubated with 1 mg/mL pooled human liver microsomes for 60 min in the absence (**A**) or presence (**B**) of the β-NADPH-regenerating system (NGS). Synthesis of AHC metabolites by recombinant cDNA-expressed CYP3A4 after incubation at 37 °C for 60 min (**C**).

To identify the phase I metabolites of AHC in HLMs, 10 µM AHC was incubated in pooled HLMs without or with the NGS for 60 min. The total ion chromatogram showed that when AHC was incubated in HLMs in the presence of the NGS, AHC was reduced. AHC decreased rapidly with the same trend as the metabolic stability results, and peaks presumed to be metabolites were generated (Figure S1). The extracted ion chromatograms of the generated AHC metabolites are shown in Figure 2A,B. AHC was detected as a deprotonated ion, [M − H]^−^, at *m*/*z* 407 and 15.6 min in the negative ion mode. All compounds presented a stronger intensity in the negative ion mode than that in the positive mode. After incubation for 60 min in the presence of the NGS, 20 metabolites were produced; deprotonated ions were observed at *m*/*z* 405 (**M1**–**M3**) by dehydrogenation at 15.1–15.8 min, *m*/*z* 421 (**M4**–**M10**) by dehydrogenation and oxidation at 13.0–15.3 min, *m*/*z* 423 (**M11**–**M13**) by mono-oxidation at 13.3–14.9 min, and *m*/*z* 439 by dioxidation (**M14**–**M20**) at 12.1–14.0 min.

### 3.3. Interpretation of Metabolite Structure

**Figure 3:**
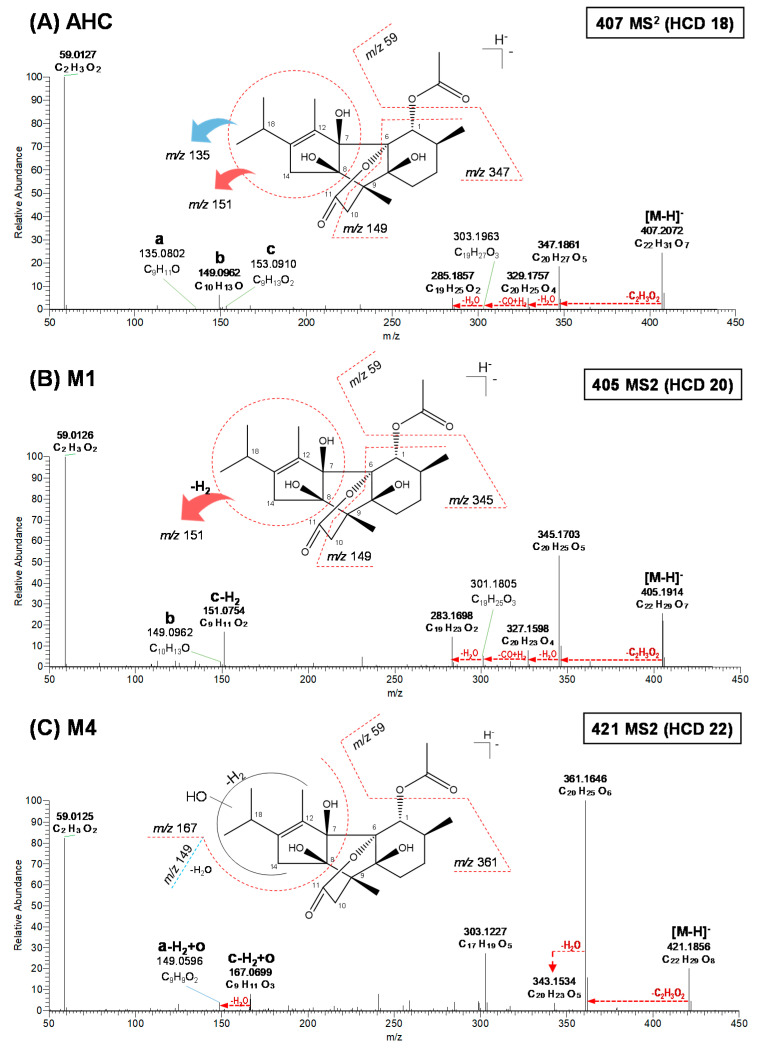

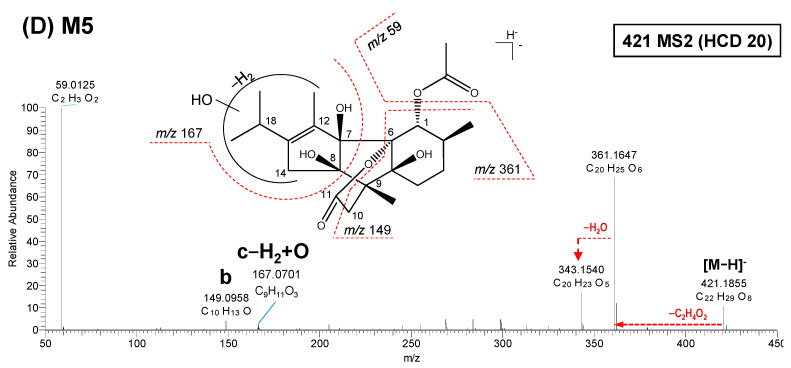
MS/MS spectra of deprotonated anhydrocinnzeylanine (AHC) (**A**), **M1** (**B**), **M4** (**C**), and **M5** (**D**), obtained using a high-resolution quadrupole-orbitrap mass spectrometer (continued). HCD, higher-energy C-trap dissociation.

To elucidate the chemical structure of the metabolites, we performed HR-MS/MS analysis of AHC and its metabolites using a quadrupole-orbitrap MS. The fragment ions of AHC and its metabolites were indicated in their elemental composition within a mass error of <5 ppm (Table S1). To identify the metabolite structure, the fragment pattern of the parent compound, i.e., AHC, was analyzed (Figure 3A). The MS spectrum of deprotonated AHC was detected at *m*/*z* 407.2072 (C_22_H_31_O_7_) in the negative ion mode; the spectra of AHC showed eight product ions. Of these ions, three major product ions served as markers of fragment ions for metabolite identification. Three key product ions were observed at *m*/*z* 135.0802 (C_9_H_11_O), 149.0962 (C_10_H_13_O), and 153.0910 (C_9_H_13_O_2_). The spectra of *m*/*z* 135 and 153 indicated the loss of the isopropyl-methylcylopentane moiety to -H_2_O (−272 Da) and the isopropyl-methylcylopentane (−254 Da) moiety, respectively. The structure of *m*/*z* 149 indicated the breakage of bonds of C6 and C7, bonds of C8 and C9, bonds of C10 and C11, and part of C1. The major ions were denoted as symbols: *m*/*z* 135 as “a”, *m*/*z* 149 as “b”, and *m*/*z* 153 as “c”, to simplify the identification of subsequent metabolite structures.

Additionally, although not providing information on metabolite structure determination, fragment ions that were derived by cleavage of the hydroxy groups in the parent existed. The product ions at *m*/*z* 347.1861 (C_20_H_27_O_5_), 329.1757 (C_20_H_25_O_4_), 303.1963 (C_19_H_27_O_3_), 285.1857 (C_19_H_25_O_2_), and 59.0127 (C_2_H_3_O_2_) showed loss of C_2_H_4_O_2_ from AHC, loss of H_2_O from *m*/*z* 347.1861, loss of CO and gain of H_2_ from *m*/*z* 329.1757, loss of H_2_O from *m*/*z* 303.1963, and loss of the carbonyl moiety, respectively.

**M1**, **M2**, and **M3** were observed at *m*/*z* 405.1914–405.1915 (C_22_H_29_O_7_), which were 2 Da (-H_2_) less than deprotonated AHC, indicating dehydrogenation of the parent ion (Table S1). The MS^2^ patterns of **M1** and **M2** were the same. **M1** and **M2** produced seven common product ions at *m*/*z* 345.1703, 327.1598, 301.1805, 283.1698, 151.0754, 149.0962, and 59.0126 (Figure 3B and Figure S2A). Five product ions at *m*/*z* 345.1703 (C_20_H_25_O_5_), 327.1598 (C_20_H_23_O_4_), 301.1805 (C_19_H_25_O_3_), 283.1698 (C_19_H_23_O_2_), and 59.0126 (C_2_H_3_O_2_) yielded the loss of C_2_H_4_O_2_ from AHC, loss of H_2_O from *m*/*z* 345.1703, loss of CO and gain of H_2_ from *m*/*z* 327.1598, loss of H_2_O from *m*/*z* 301.1805, and loss of the carbonyl moiety, respectively. Another two major product ions of **M1** and **M2** were observed at *m*/*z* 151.0754 (c-H_2_) and *m*/*z* 149.0962 (b). The product ion at *m*/*z* 151 was formed by dehydrogenation at the isopropyl-methylcylopentane moiety, and the fragment at *m*/*z* 149 was the same as that observed in deprotonated AHC.

The fragment ions of **M3** were observed at *m*/*z* 363.1809, 345.1704, 327.1598, 247.0972, 219.1020, 203.1070, 151.0756, 149.0961, and 59.0127 (Figure S2B). In **M3**, the same product ions at *m*/*z* 151.0756 and 149.0961 as those in **M1** and **M2** were observed, indicating dehydrogenation at the isopropyl-methylcylopentane moiety. Although exact structural analysis was not possible to identify, three product ions were observed in **M3** that differed from those in **M1** and **M2**. The product ions at *m*/*z* 219.1020 (C_13_H_15_O_3_) and 203.1070 (C_13_H_15_O_2_) indicated the loss of CO from 247.0972 and the loss of O from *m*/*z* 219.1020, respectively. The product ion at *m*/*z* 219.1020 was identified with breakage of the bond between C6 and C7, the bond between C5 and C9, and the single bond between oxygen and C11, and with dehydrogenation at the isopropyl-methylcylopentane moiety. Therefore, **M1**–**M3** were dehydrogenated metabolites in the isopropyl-methylcylopentane moiety.

**M4**–**M10** were observed at *m*/*z* 421.1852–421.1855 (C_22_H_29_O_8_), which were 14 Da (-H_2_ + O) larger than deprotonated AHC, indicating the dehydrogenation and oxidation of the parent ion (Table S1). The MS^2^ patterns of **M4** and **M9** were the same (Figure 3C and Figure S3A). The product ions of **M4** were detected at *m*/*z* 361.1646 (C_20_H_25_O_6_), 343.1534 (C_20_H_23_O_5_), 167.0699 (C_9_H_11_O_3_), 149.0596 (C_9_H_9_O_2_), and 59.0125 (C_2_H_3_O_2_) (Figure 3C). Two characteristic fragment ions at *m*/*z* 167 (c-H_2_ + O) and 149 (a-H_2_ + O) were formed by dehydrogenation and oxidation at the isopropyl-methylcylopentane moiety; *m*/*z* 149 did not represent the b part in AHC but was a product ion derived from *m*/*z* 167. Another three product ions at *m*/*z* 361.1646 (C_20_H_25_O_6_), 343.1534 (C_20_H_23_O_5_), and 59.0125 (C_2_H_3_O_2_) produced the loss of C_2_H_4_O_2_ from the deprotonated precursor ion of **M4** and **M9**, the loss of H_2_O from *m*/*z* 361, and the loss of the carbonyl moiety, respectively.

**M5** and **M10** had the same MS^2^ patterns (Figure 3D and Figure S3B). The product ions of **M5** were detected at *m*/*z* 361.1647 (C_20_H_25_O_6_), 343.1540 (C_20_H_23_O_5_), 167.0701 (C_9_H_11_O_3_), 149.0958 (C_10_H_13_O), and 59.0125 (C_2_H_3_O_2_). Two major product ions at *m*/*z* 149.0958 (C_10_H_13_O) and 167.0701 (C_9_H_11_O_3_) were confirmed. The product ion at *m*/*z* 149 (b) was the same as that observed in deprotonated AHC. In contrast, the fragment at *m*/*z* 167 (c-H_2_ + O) was formed by dehydrogenation and oxidation at the isopropyl-methylcylopentane moiety.

**M6**, **M7**, and **M8** showed the same MS^2^ patterns (Figure S3C–E). **M6** produced product ions at *m*/*z* 361.1646 (C_20_H_25_O_6_), 343.1541 (C_20_H_23_O_5_), 235.0966 (C_13_H_15_O_4_), 167.0700 (C_9_H_11_O_3_), 149.0594 (C_9_H_9_O^2^), and 59.1025 (C_2_H_3_O_2_). Similar to **M4** and **M9**, the fragment ions at *m*/*z* 167.0700 (c-H_2_ + O) and 149.0594 (a-H_2_ + O) in **M6** indicated dehydrogenation and oxidation at the isopropyl-methylcylopentane moiety. Additionally, the MS/MS of **M6**–**M8** showed a common specific product ion at *m*/*z* 235, indicating bond breakage between C6 and C7, between C5 and C9, and between oxygen and C11. Therefore, **M4**–**M10** were dehydrogenated and oxidated metabolites in the isopropyl-methylcylopentane moiety.

**Figure 4:**
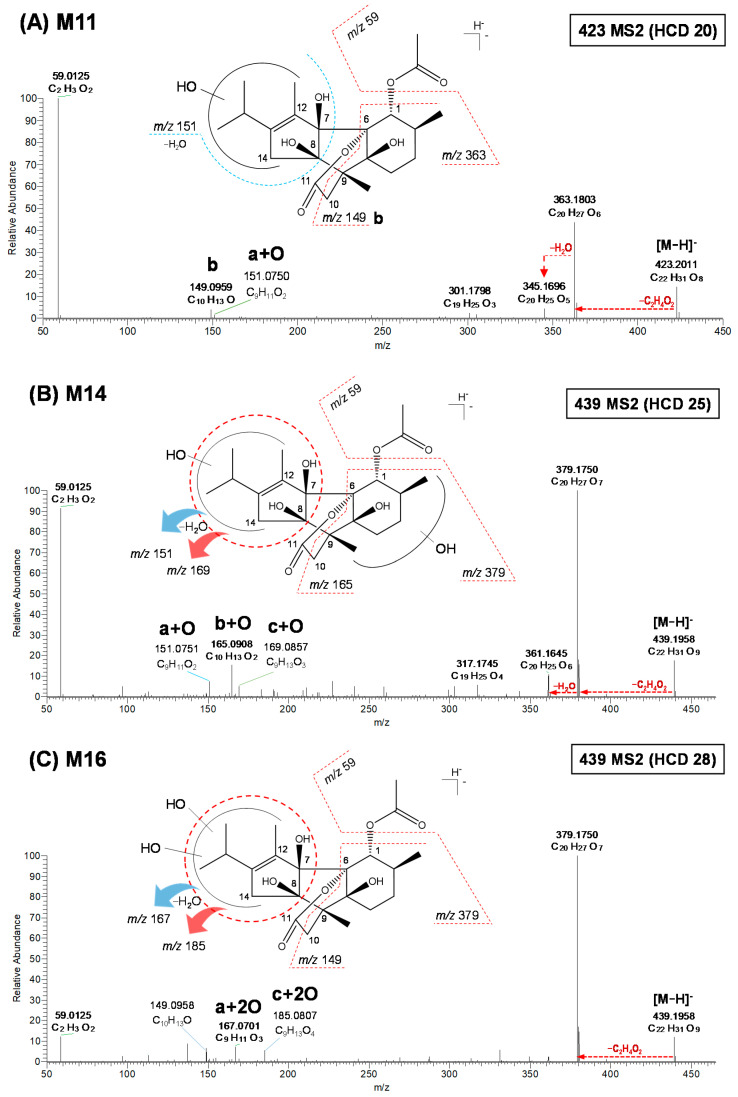
MS/MS spectra of deprotonated **M11** (**A**), **M14** (**B**), and **M16** (**C**) obtained using a high-resolution quadrupole-orbitrap mass spectrometer. HCD, higher-energy C-trap dissociation.

**M11**, **M12**, and **M13** were observed at *m*/*z* 423.2010–423.2011 (C_22_H_31_O_8_), which were 16 Da (+O) larger than deprotonated AHC, indicating mono-oxidation of the parent ion (Table S1). **M11**–**M13** exhibited similar MS^2^ fragment patterns (Figure 4A and Figure S4A,B). The product ions of **M11** at *m*/*z* 363.1803 (C_20_H_27_O_6_), 345.1697 (C_20_H_25_O_5_), 151.0705 (C_9_H_11_O_2_), 149.0959 (C_10_H_13_O), and 59.0125 (C_2_H_3_O_2_) formed the loss of C_2_H_4_O_2_ from AHC, loss of H_2_O from *m*/*z* 345, and loss of the carbonyl moiety. These three metabolites had two common characteristic fragment ions at *m*/*z* 151 (a + O) and 169 (c + O), indicating mono-oxidation at isopropyl-methylcylopentane. Individually, **M11** showed *m*/*z* 151, **M12** showed *m*/z 169, and **M13** showed *m*/*z* 151 and 161. Additionally, one common ion at *m*/*z* 149 was detected in all three metabolites, indicating the b part of AHC. Therefore, **M11**–**M13** were mono-oxidated metabolites in the isopropyl-methylcylopentane moiety.

**M14**–**M20** were observed at *m*/*z* 439.1956–439.1958 (C_22_H_31_O_9_), which were 32 Da (+O_2_) larger than deprotonated AHC, indicating dioxidation of the parent ion (Table S1). The seven dioxidative metabolites can be divided into two groups according to their oxidation location. First, **M14**, **M15**, and **M19** showed similar MS^2^ fragment patterns (Figure 4B and Figure S5). Second, the product ions of **M14** were detected at *m*/*z* 379.1750 (C_20_H_27_O_7_), 361.1645 (C_20_H_25_O_6_), 169.0857 (C_9_H_13_O_3_), 165.0908 (C_10_H_13_O_2_), 151.0751 (C_9_H_11_O_2_), and 59.0125 (C_2_H_3_O_2_). The characteristic fragment ions at *m*/*z* 169.0857 (c + O) and 151.0751 (a + O) were formed by mono-oxidation at the isopropyl-methylcylopentane moiety and by the loss of H_2_O from *m*/*z* 169, respectively. The product ions at *m*/*z* 165 showed another mono-oxidation position representing the oxidation of the b moiety at positions C3, C4, C10, C15, or C16. The exact location of each oxidation could not be confirmed due to limitations in interpreting the MS^2^ spectra.

The second group of dioxidative metabolites was **M16**, **M17**, **M18**, and **M20** (Table S1), which had the same MS^2^ patterns (Figure 4C and Figure S6). The product ions of **M16** were observed at *m*/*z* 379.1750 (C_20_H_27_O_7_), 185.0807 (C_9_H_13_O_4_), 167.0701 (C_9_H_11_O_3_), 149.0958 (C_10_H_13_O), and 59.0125 (C_2_H_3_O_2_). The major fragment ions at *m*/*z* 185.0807 (c + 2O) and 167.0701 (a + 2O) were yielded by dioxidation at the isopropyl-methylcylopentane moiety and by loss of H_2_O from *m*/*z* 185, respectively. The product ion at *m*/*z* 149 was the same as that identified in deprotonated AHC. Finally, **M16**, **M17**, **M18**, and **M20** were dioxidated metabolites in the isopropyl-methylcylopentane moiety.

### 3.4. Time-Dependent Formation of AHC Metabolites

**Figure 5:**
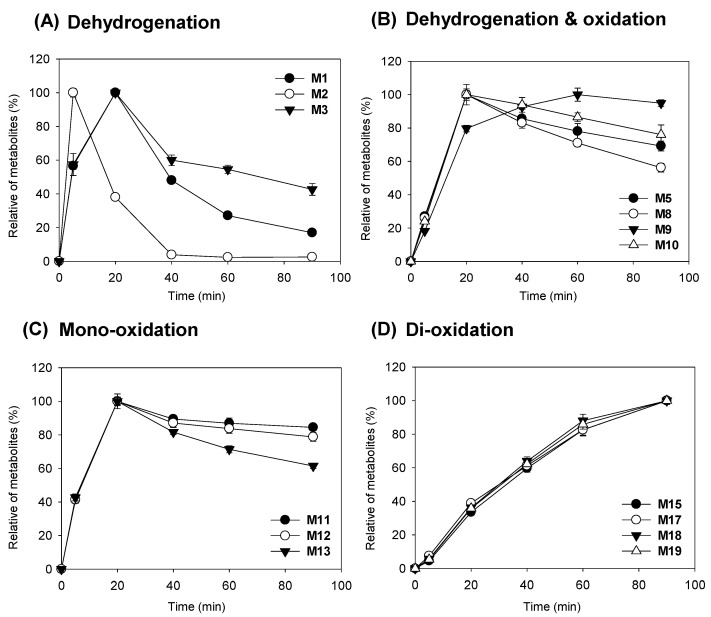
Time-dependent synthesis of anhydrocinnzeylanine (AHC) metabolites. Dehydrogenated metabolites (**M1**, **M2**, and **M3**) (**A**), dehydrogenated and oxidated metabolites (**M5**, **M8**, **M9**, and **M10**) (**B**), mono-oxidated metabolites (**M11**, **M12**, and **M13**) (**C**), and dioxidated metabolites (**M15**, **M17**, **M18**, and **M19**) (**D**) were incubated with human liver microsomes at 37 °C for 0–90 min in the presence of the β-NADPH-regenerating system. Bars indicate standard error (*n* = 3).

To identify the time-dependent metabolism of AHC in HLMs, we incubated AHC with pooled HLMs in the presence of the NGS for 0, 5, 20, 40, 60, and 90 min (Figure 5). The hydrogenated metabolites (**M1**–**M3**) showed a sharp increase in the initial production of the reaction but decreased as the reaction time increased, indicating their further conversion into other metabolites after the hydrogenation of AHC. The hydrogenated and oxidative metabolites (**M5**, **M8**, **M9**, and **M10**) tended to gradually decrease after increasing continuously up to 20 min, except for **M5**, which continued to increase depending on the reaction time. By comparing the relative production over time, it was revealed that AHC was first metabolized by hydrogenation, followed by oxidation. Considering the oxidative metabolites of AHC, the mono-oxidative metabolites (**M11**, **M12**, and **M13**) peaked at 20 min and then decreased with the reaction time, whereas the dioxidative metabolites (**M15**, **M17**, **M18**, and **M19**) increased continuously depending on the reaction time.

### 3.5. Characterization of AHC Metabolism in cDNA-Expressed Recombinant CYP Isoforms

To confirm the metabolism of AHC by CYP enzymes, AHC was incubated in HLMs with SKF-525A, i.e., a nonspecific CYP inhibitor (Figure S7). The metabolism of AHC was inhibited, and the residual amount of AHC after the reaction was significantly increased depending on the SKF-525A treatment concentration; thus, AHC was metabolized in a CYP-dependent manner. Consequently, as the metabolism of AHC was inhibited by SKF-525A treatment, the production of all metabolites was reduced in an SKF-525A concentration-dependent manner.

**Figure 6:**
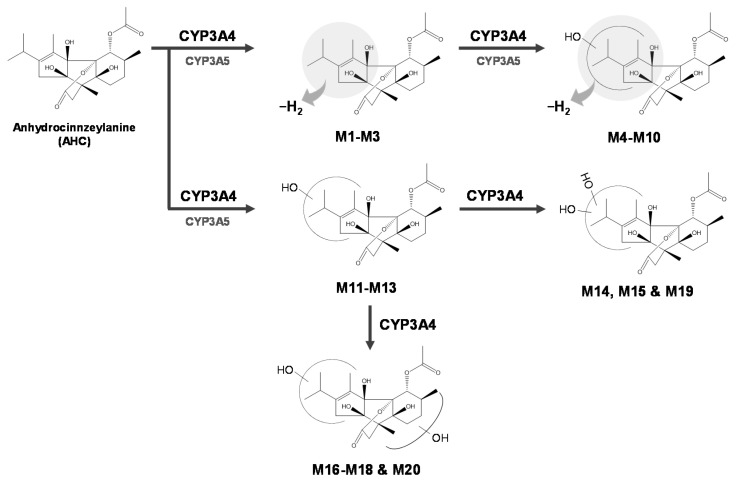
Postulated metabolic pathway of anhydrocinnzeylanine in human liver microsomes.

To identify the CYP isoforms involved in AHC metabolism, AHC (10 µM) was incubated with eleven cDNA-expressed recombinant CYP isoforms (CYP1A1, CYP1A2, CYP2A6, CYP2B6, CYP2C8, CYP2C9, CYP2C19, CYP2D6, CYP2E1, CYP3A4, and CYP3A5) in the presence of the NGS. As a result, CYP3A4 produced **M1**–**M20** as the major CYP responsible for the metabolism of AHC in HLMs (Figure 2C). Additionally, some metabolites such as **M1**, **M2**, **M8**, **M11**, **M12**, and **M13** were generated at low levels by CYP3A5 (Figure S8). However, the metabolites of AHC were not observed from reactions with other CYPs. Thus, in the metabolism of AHC in HLMs, CYP3A4 was involved as the main enzyme, and four types of metabolites were generated. Based on these results, the postulated metabolic pathway of AHC in the HLMs is summarized in Figure 6.

## 4. Discussion

Diterpene compounds are known to exhibit excellent biological activity as active substances in several medicinal plants, and research is underway to use diterpene compounds as pharmaceuticals or health functional foods [20]. Although phase I and phase II metabolism experiments were conducted to develop diterpene compounds of several skeletal types as new drug candidates, metabolism studies of diterpene compounds with the same backbone as AHC have not been reported. In this study, the metabolism of seven diterpenoids isolated from *C. cassia* was studied in HLMs. Of the seven compounds, four were metabolically stable; however, anhydrocinnzeylanol and cinnzeylanone were decreased by approximately 20%, whereas AHC showed a unique reduction in metabolic stability in HLMs (Figure 1B). Although the compounds have a common structure, i.e., the (3aR,3bR,5S,7aS,8R,8aR)-2-isopropyl-3,5,8-trimethyldecahydro-1H-3b,8(epoxy-ethano)-cyclopenta[a]indene-3a,7a,8a-triol structure, the rapid metabolism of AHC can be explained by structural differences relative to the other six compounds [21,22]. Cinncassiol B, cinnzeylanol, cinnzeylanone, and cinnzeylanine have C11–C12 bond, C13 hydroxylation and C11 hydroxylation structures in common. These similarities are suggested to be related to the enhanced metabolic stability, unlike that observed for AHC. For cinncassiol A, C19 hydroxylation enhanced the metabolic stability, whereas anhydrocinnzeylanol, in particular, differs from AHC in that C1 does not have an acetyl group. C1 acetylation plays an important role in the metabolism of AHC. Anhydrocinnzeylanol, which has an AHC-like structure, except for C1-acetyl, is metabolized by 20%, whereas AHC with the C1-acetyl group is metabolized by 95% under the same conditions. In addition, the C1-acetyl moiety was not directly metabolized, i.e., we did not identify the O-deacetyl metabolite of AHC in HLMs. Thus, the C1-acetyl group is predicted to be important for interactions between AHC and CYP enzymes, especially CYP3A4, in HLMs.

In this study, AHC was predominantly metabolized by CYP3A4 following incubation with cDNA-expressed recombinant CYP3A4 in the presence of the NGS. All metabolites (**M1**–**M20**) were formed in CYP3A4, similar to that in HLMs (Figure 2C). In addition, some metabolites, i.e., **M1**, **M2**, **M8**, **M11**, **M12**, and **M13**, were also detected in small amounts in CYP3A5. CYP3A is the most abundant enzyme in the liver, constituting approximately 30% of all CYP proteins in the liver [23]. Considering that CYP3A oxidizes 40%–50% of drugs, it is of great importance in metabolic reactions and drug–drug interactions [24]. CYP3A4 is the dominant CYP, accounting for 80% of CYPs in small intestinal enterocytes, and is an important factor in the bioavailability of CYP3A4 substrate drugs [25]. Although the rate of metabolism of AHC in human intestinal microsomes was not evaluated in this study, the effect of intestinal CYP3A4-mediated biotransformation should be considered when evaluating the in vivo bioavailability of AHC.

Notably, the dehydrogenation of −2 Da in the parent compound showed that large amounts of metabolites were rapidly produced at the start of the reaction (Figure 5). Thus, the dehydrogenation reaction may involve enzymes other than CYPs. The major oxidative enzymes other than CYPs involved in the metabolism of drugs and other xenobiotics include flavin-containing monooxygenases, molybdenum hydroxylases (aldehyde oxidase and xanthine oxidase), prostaglandin H synthase, lipoxygenases, amine oxidases (monoamine, polyamine, diamine, and semicarbazide-sensitive amine), alcohol dehydrogenases, and aldehyde dehydrogenase [26]. However, the yield of all metabolites, including dehydrogenated metabolites, was significantly decreased following the addition of SKF-525A. The metabolites were synthesized by cDNA-expressed recombinant CYP3A4, suggesting that CYP3A4 is the main metabolizing enzyme for AHC dehydrogenation and oxidation in HLMs. In a previous study, the dehydrogenation of many compounds by CYP and the chemical mechanism of dehydrogenation were reported in detail [27]. Furthermore, it has been confirmed that testosterone is dehydrogenated by CYP3A, resulting in the formation of the metabolite 6-dehydrotestosterone [21,22]. Moreover, CYP3A4 mediates the dehydrogenation of diverse compounds such as raloxifene and ezlopitant [28,29,30]. This study did not determine exactly where dehydrogenation occurred in the isopropyl-methylcylopentane moiety of **M1**–**M3**, but dehydrogenated metabolites were generated by CYP3A.

Although we focused on identifying the phase I metabolites of seven diterpenes, including AHC, there is a possibility that phase II metabolism may proceed from a structural viewpoint, such as having -OH groups. Therefore, phase II metabolism studies should be conducted to understand the entire metabolic pathway of diterpene compounds.

## 5. Conclusions

We verified that AHC is metabolized to three dehydrogenated metabolites (**M1**–**M3**), seven dehydrogenated and oxidated metabolites (**M4**–**M10**), three mono-oxidated metabolites (**M11**–**M13**), and seven dioxidated metabolites (**M14**–**M20**) in HLMs, in which CYP3A4 was involved in the synthesis of all metabolites. Additionally, the structures of the metabolites were determined using HR/high-accuracy MS/MS. Finally, the possible metabolic fate of AHC in HLMs was summarized (Figure 6).

## Data Availability

Not applicable.

## Supplementary Materials

The following are available online at https://www.mdpi.com/article/10.3390/pharmaceutics13081316/s1, Figure S1: Total ion chromatograms (TIC) for anhydrocinnzeylanine (AHC) and its metabolites. AHC (10 µM) was incubated with 1 mg/mL of pooled human liver microsomes for 60 min in the absence (**A**) or presence (B) of the β-NADPH-regenerating system (NGS). Figure S2: MS/MS spectra of dehydrogenated **M2** (A) and **M3** (B) using a high-resolution quadrupole-orbitrap mass spectrometer. Figure S3: MS/MS spectra of dehydrogenated **M9** (A), **M10** (B), **M6** (C), **M7** (D), and **M8** (E) using a high-resolution quadrupole-orbitrap mass spectrometer. Figure S4: MS/MS spectra of dehydrogenated **M12** (A) and **M13** (B) using a high-resolution quadrupole-orbitrap mass spectrometer. Figure S5: MS/MS spectra of dehydrogenated **M15** (A) and **M19** (B) using a high-resolution quadrupole-orbitrap mass spectrometer. Figure S6: MS/MS spectra of dehydrogenated **M17** (A), **M18** (B), and **M20** (C) using a high-resolution quadrupole-orbitrap mass spectrometer. Figure S7: Effect of a nonspecific CYP inhibitor, SKF 525-A, in the metabolism of AHC in HLMs. Inhibition of AHC metabolism following SKF-525 treatment (0, 0.25, and 1.25 mM) (A). Decreased production of **M1**, **M2**, and **M3** (B); **M4**–**M10** (C); **M11**–**M13** (D); and **M14**–**M20** (E). The data are expressed as means ± standard errors (SE) of the triplicate samples. Figure S8: Formation of AHC metabolites in recombinant cDNA-expressed CYP3A5 after incubation at 37 °C for 60 min. Table S1: Elemental composition of key product ions of anhydrocinnzeylanine and its metabolites in human liver microsomes using high-resolution quadrupole-orbitrap mass spectrometry.
