# Supplementary material for: Metabolism of Diterpenoids Derived from the Bark of Cinnamomum cassia in Human Liver Microsomes
Source: Pharmaceutics. 2021 Aug 23;13(8):1316. doi: 10.3390/pharmaceutics13081316 (PMC8400920; doi:10.3390/pharmaceutics13081316)
Supplement: Supplementary file 1 [file pharmaceutics-13-01316-s001.zip › pharmaceutics-1330050-SI.pdf]

pharmaceutics

MDPI

Supplementary Materials: Metabolism of Diterpenoids Derived from the Bark of *Cinnamomum cassia* in Human Liver Microsomes

Su Min Choi, Van Cong Pham, Sangkyu Lee and Jeong Ah Kim

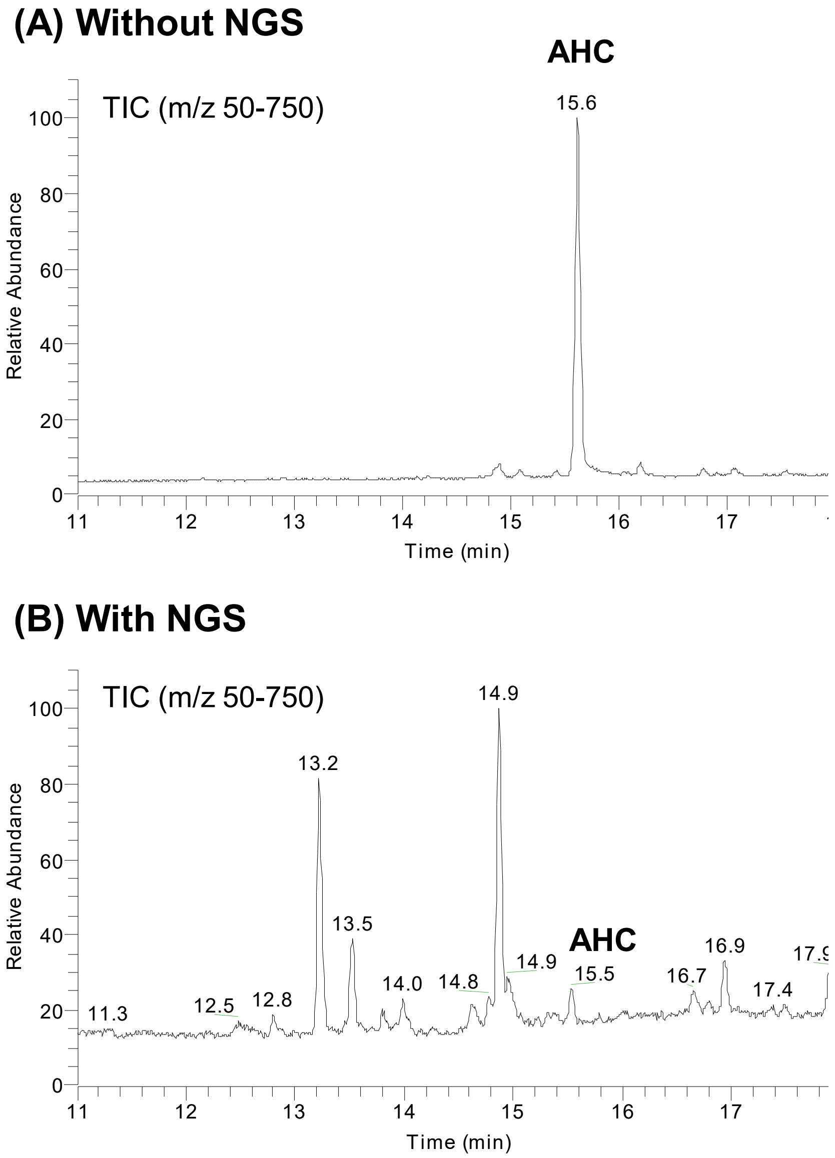

Figure S1. Total ion chromatograms (TIC) for anhydrocinnzeylanine (AHC) and its metabolites. AHC (10 μM) was incubated with 1 mg/mL of pooled human liver microsomes for 60 min in the absence (**A**) or presence (**B**) of a β-NADPH-regenerating system (NGS).

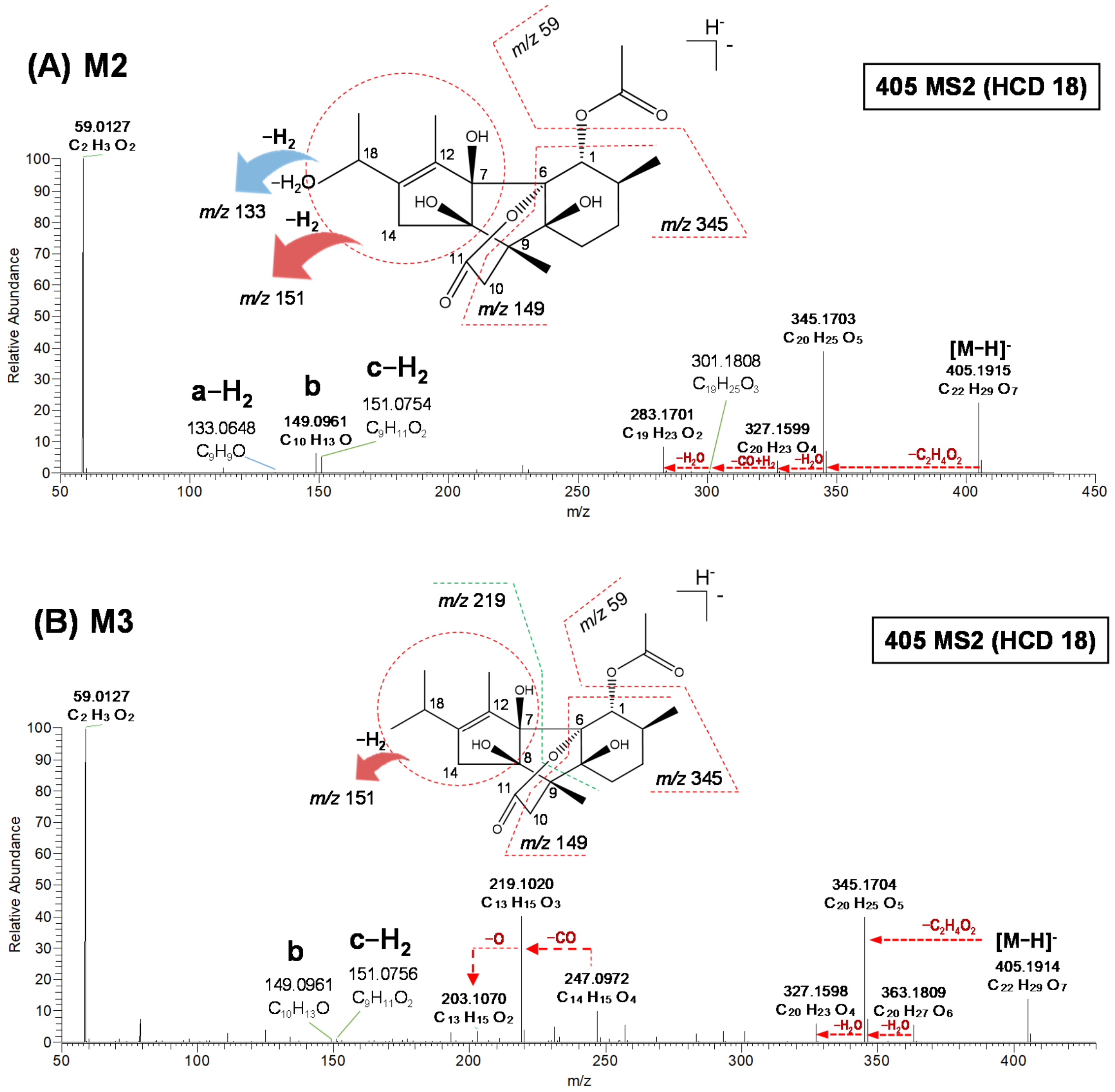

Figure S2. MS/MS spectra of dehydrogenated M2 (**A**) and M3 (**B**) using a high-resolution quadrupole-orbitrap mass spectrometer.

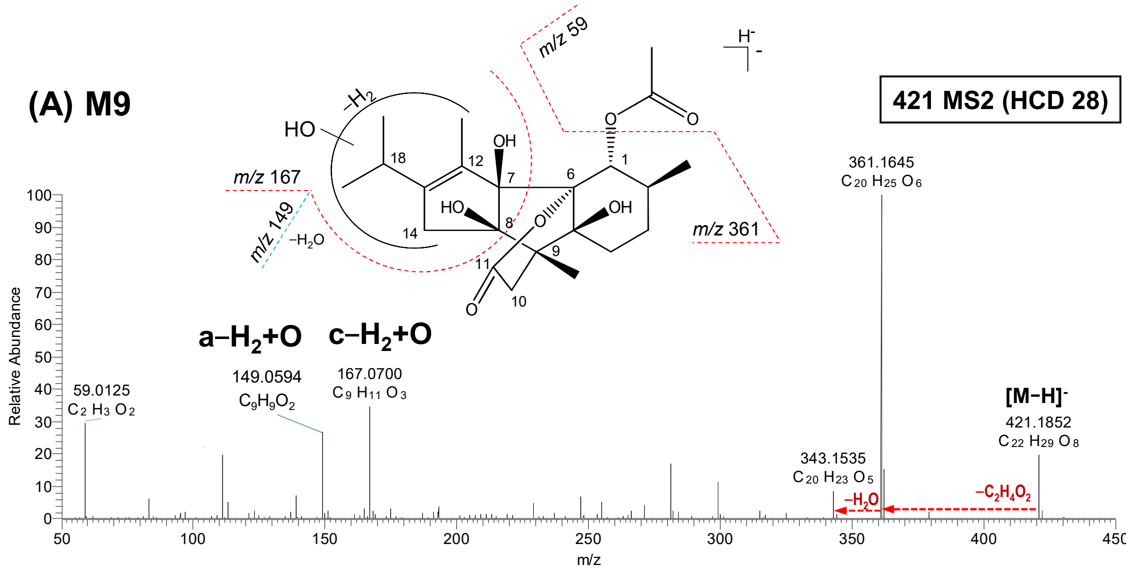
(A) M9
421 MS2 (HCD 28)
m/z 59
m/z 167
m/z 149
-H2O
-H2
m/z 361
a–H2+O
c–H2+O
59.0125
C2 H3 O2
149.0594
C9H9O2
167.0700
C9 H11 O3
343.1535
C20 H23 O5
361.1645
C20 H25 O6
[M–H]-
421.1852
C22 H29 O8
–H2O
–C2H4O2
Relative Abundance
m/z

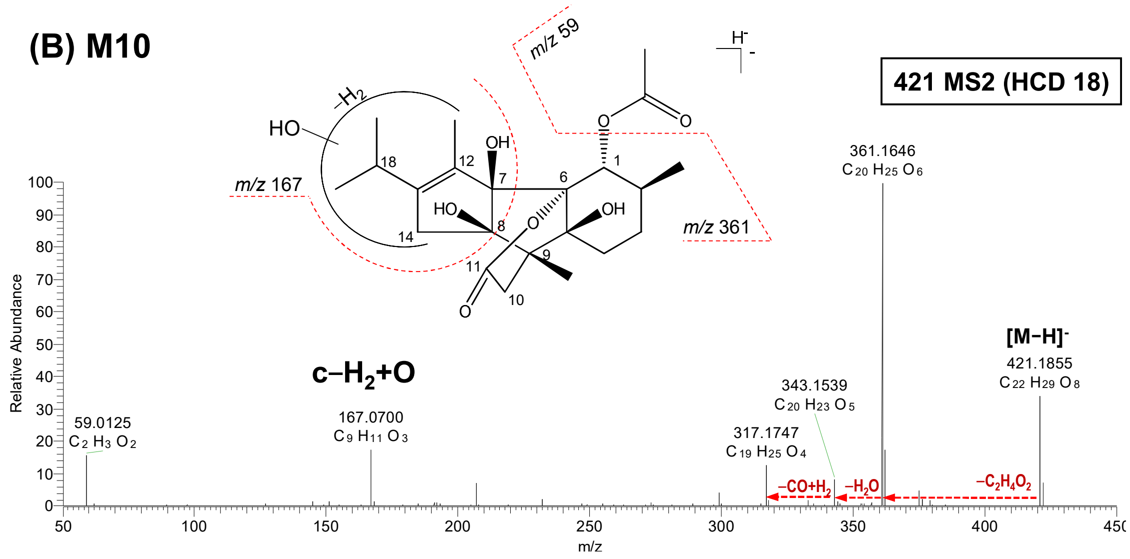
(B) M10
421 MS2 (HCD 18)
m/z 59
m/z 167
-H2
m/z 361
c–H2+O
59.0125
C2 H3 O2
167.0700
C9 H11 O3
317.1747
C19 H25 O4
343.1539
C20 H23 O5
361.1646
C20 H25 O6
[M–H]-
421.1855
C22 H29 O8
–CO+H2
–H2O
–C2H4O2
Relative Abundance
m/z

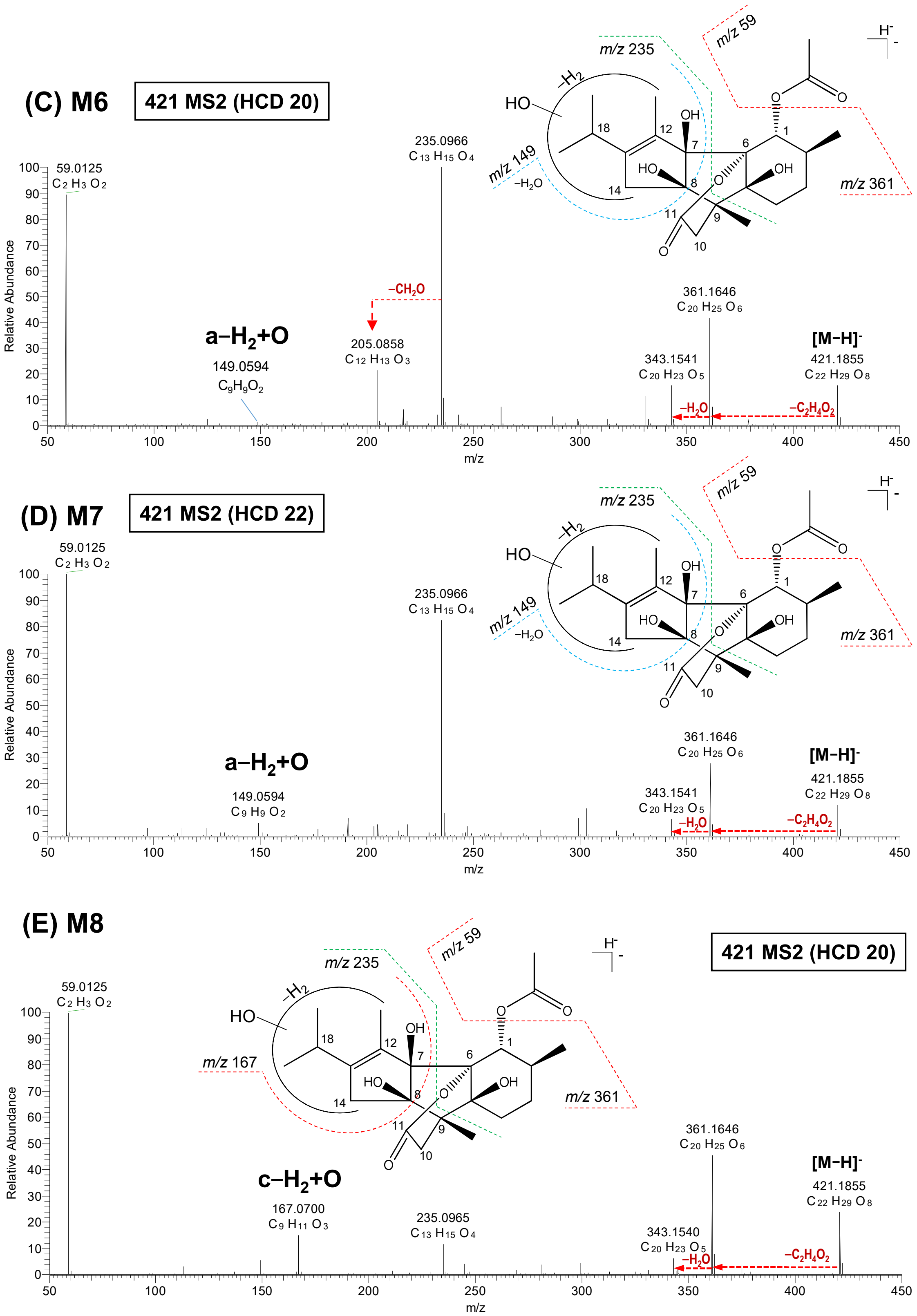

Figure S3. MS/MS spectra of dehydrogenated M9 (**A**), M10 (**B**), M6 (**C**), M7 (**D**) and M8 (**E**) using a high-resolution quadrupole-orbitrap mass spectrometer.

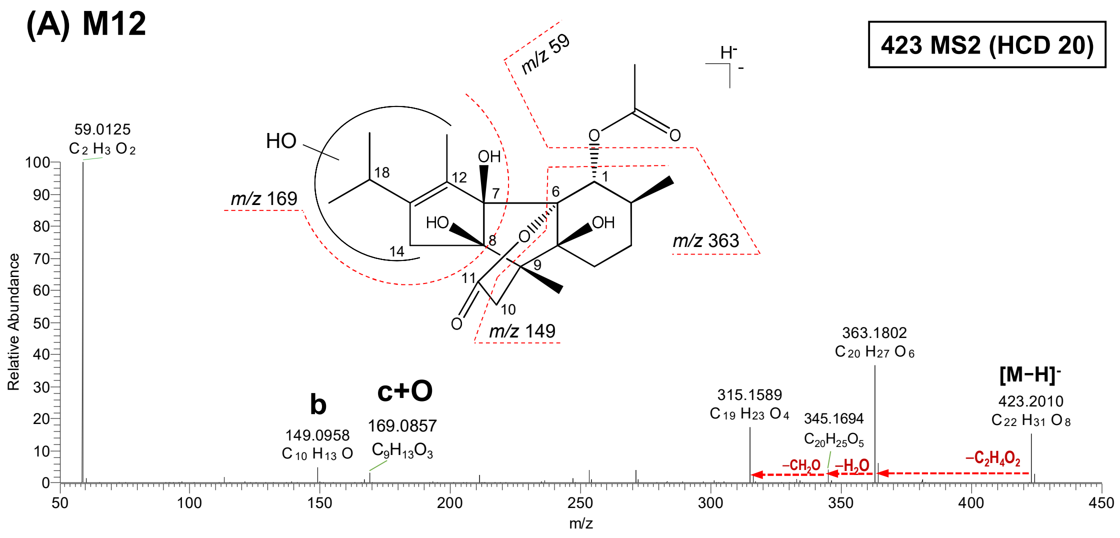

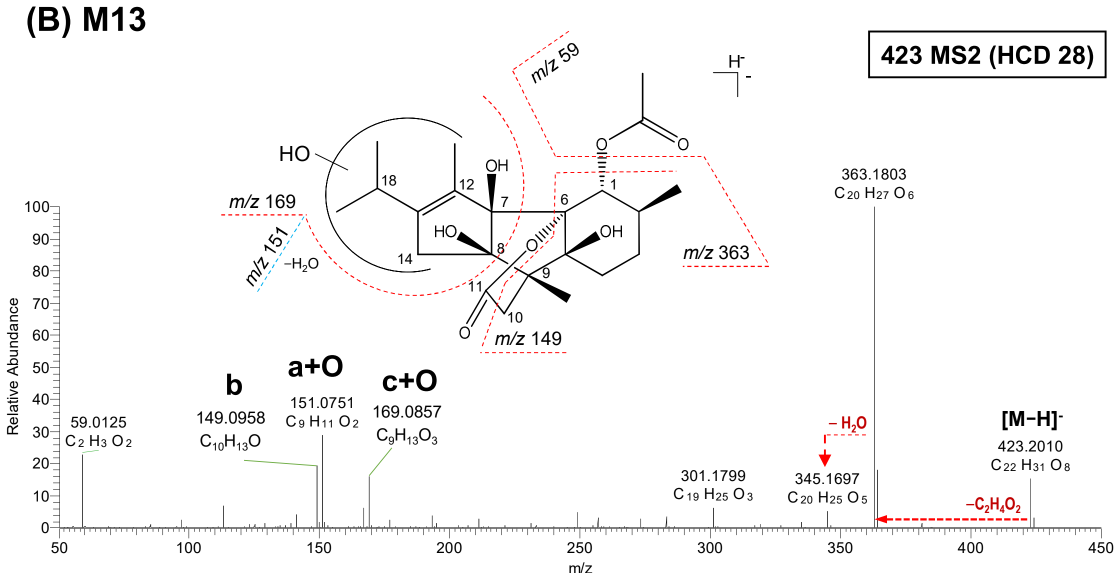

Figure S4. MS/MS spectra of dehydrogenated M12 (**A**) and M13 (**B**) using a high-resolution quadrupole-orbitrap mass spectrometer.

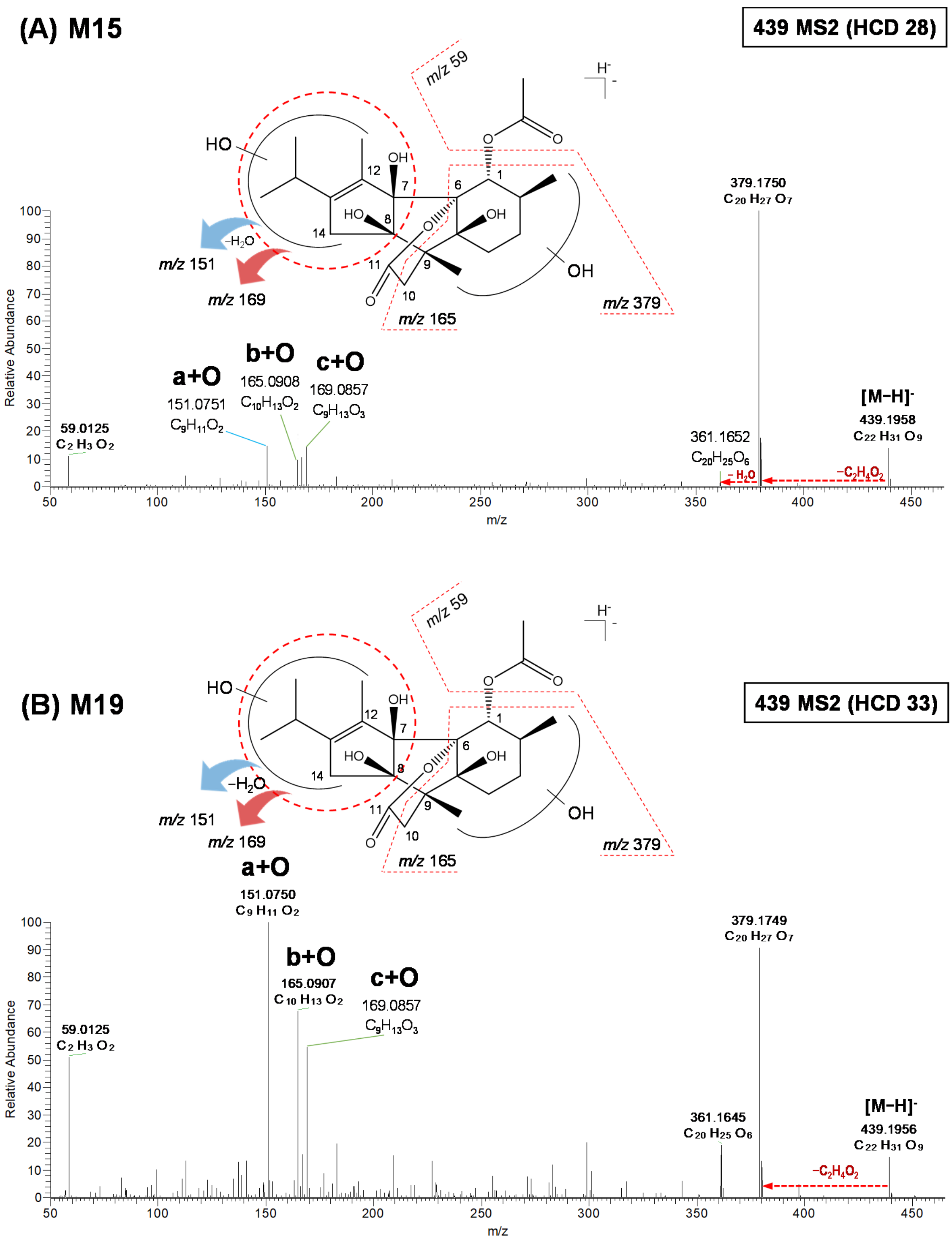

Figure S5. MS/MS spectra of dehydrogenated M15 (**A**) and M19 (**B**) using a high-resolution quadrupole-orbitrap mass spectrometer.

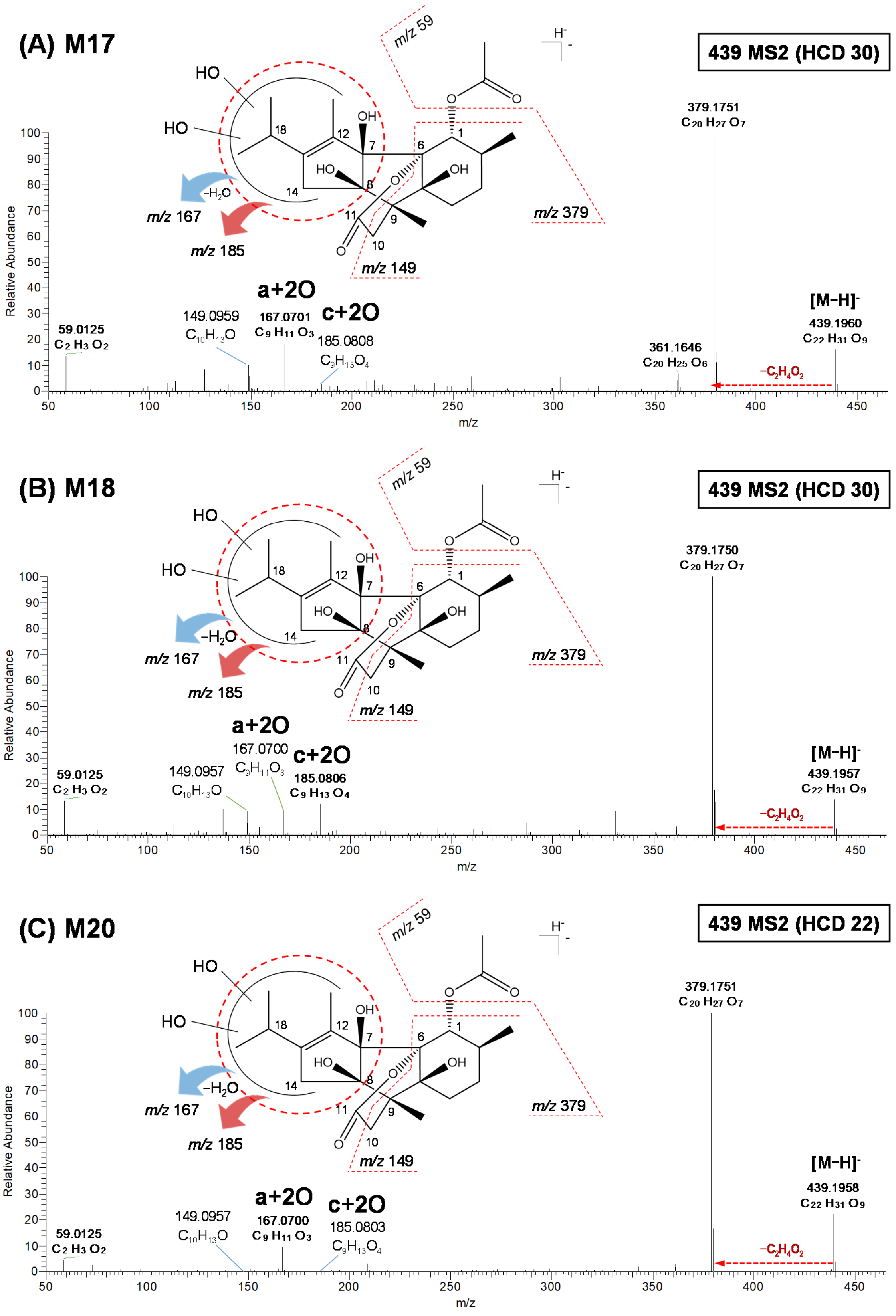

Figure S6. MS/MS spectra of dehydrogenated M17 (**A**), M18 (**B**) and M20 (**C**) using a high-resolution quadrupole-orbitrap mass spectrometer.

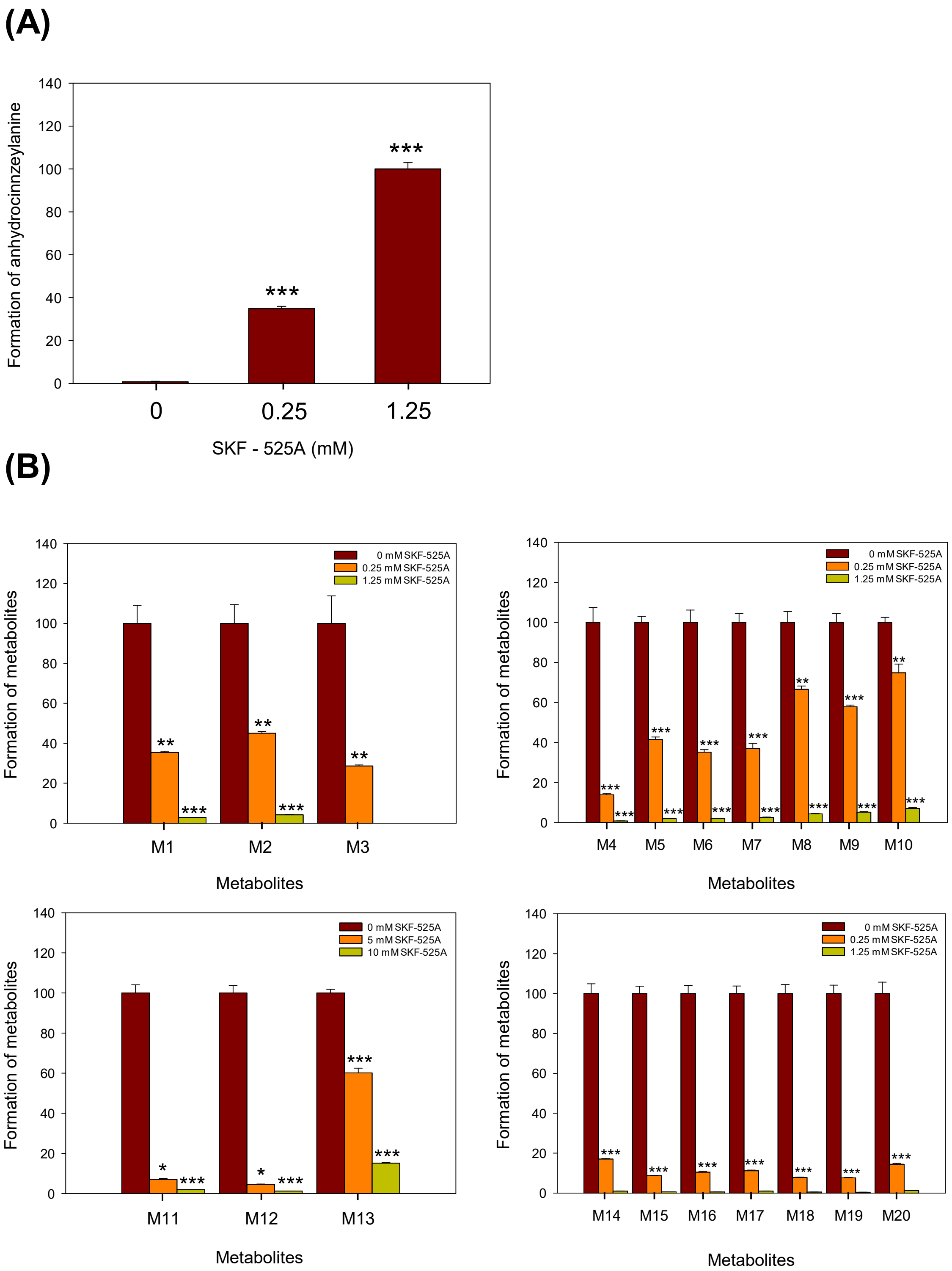

Figure S7. Effect of non-specific CYP inhibitor, SKF 525-A in AHC metabolism in HLMs. Inhibition of AHC metabolism by SKF-525 treatment (0, 0.25 and 1.25 mM) (**A**). Decreased of formation of M1, M2 and M3 (**B**), M4–M10 (**C**), M11–M13 (**D**) and M14–M20 (E). The data are expressed as mean ± standard errors (SE) of the triplicate samples. Bars indicate standard error (n = 3). * $p < 0.05$, ** $p < 0.01$ and *** $p < 0.001$ *vs* 0 mM SKF-525A.

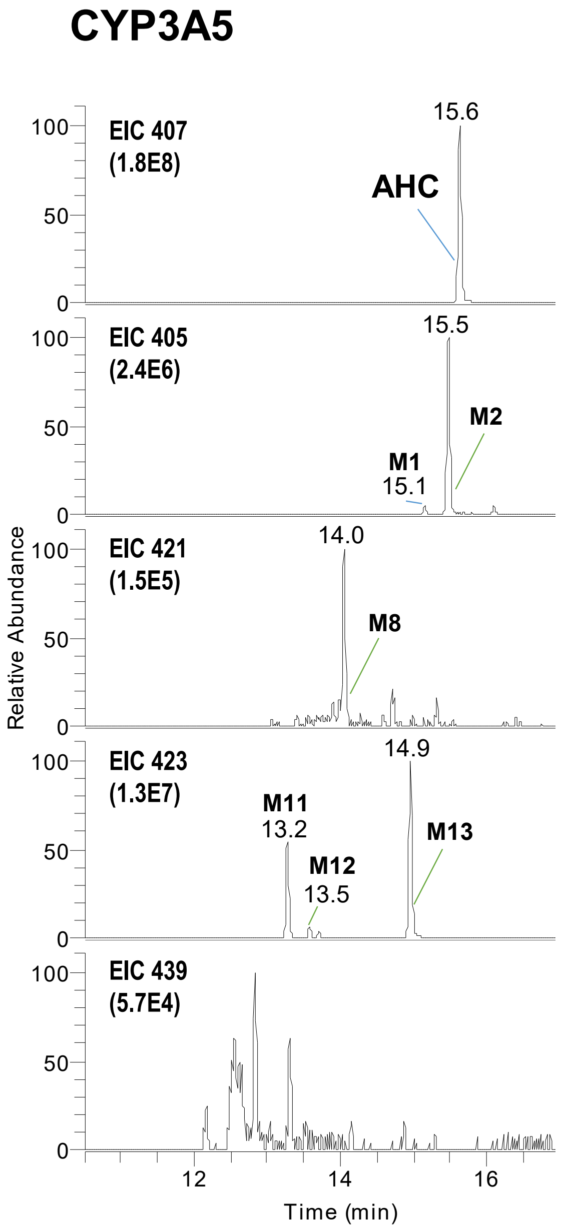

Figure S8. The formation of AHC metabolites in recombinant *cDNA-expressed* CYP3A5 after incubation at 37 °C for 60 min.

Table S1. Elemental composition of key product ions of anhydrocinnzeylanine and its metabolites in human liver microsomes using high-resolution quadrupole-orbitrap mass spectrometry.

Compound	Precursor Ions (m/z)			CE (eV)	Product Ion (m/z)	Elemental Comp. (exp.)	Error (ppm)
	[M-H]$^-$	Elemental Comp. (exp.)	Error (ppm)				
**Anhydro-cinnzeylanine**	**407.2072**	**$C_{22}H_{31}O_7$**	**1.7**	18	347.1861	$C_{20}H_{27}O_5$	2.4
				18	329.1757	$C_{20}H_{25}O_4$	2.3
				18	303.1963	$C_{19}H_{27}O_3$	2.6
				18	285.1857	$C_{19}H_{25}O_2$	2.7
				18	153.0910	$C_9H_{13}O_2$	0.0
				18	149.0962	$C_{10}H_{13}O$	0.3
				18	135.0802	$C_9H_{11}O$	0.9
				18	59.0127	$C_2H_3O_2$	−1.2
M1	405.1914	$C_{22}H_{29}O_7$	1.6	20	345.1703	$C_{20}H_{25}O_5$	2.0
				20	327.1598	$C_{20}H_{23}O_4$	2.2
				20	301.1805	$C_{19}H_{25}O_3$	2.3
				20	283.1698	$C_{19}H_{23}O_2$	2.0
				20	151.0754	$C_9H_{11}O_2$	0.1
				20	149.0962	$C_{10}H_{13}O$	0.2
				20	59.0126	$C_2H_3O_2$	−1.8
M2	405.1915	$C_{22}H_{29}O_7$	1.6	18	345.1703	$C_{20}H_{25}O_5$	2.0
				18	327.1599	$C_{20}H_{23}O_4$	2.4
				18	301.1808	$C_{19}H_{25}O_3$	3.4
				18	283.1701	$C_{19}H_{23}O_2$	2.9
				18	151.0754	$C_9H_{11}O_2$	0.1
				18	149.0961	$C_{10}H_{13}O$	0.2
				18	133.0648	C_9H_9O	−0.2
				18	59.0127	$C_2H_3O_2$	−1.6
M3	405.1914	$C_{22}H_{29}O_7$	1.5	18	363.1809	$C_{20}H_{27}O_6$	1.9
				18	345.1704	$C_{20}H_{25}O_5$	2.2
				18	327.1598	$C_{20}H_{23}O_4$	2.2
				18	247.0972	$C_{14}H_{15}O_4$	2.8
				18	219.1020	$C_{13}H_{15}O_3$	1.9
				18	203.1070	$C_{13}H_{15}O_2$	1.5
				18	151.0753	$C_9H_{11}O_2$	−0.1
				18	149.0961	$C_{10}H_{13}O$	0.0
				18	59.0127	$C_2H_3O_2$	−1.6
M4	421.1856	$C_{22}H_{29}O_8$	−0.1	22	361.1646	$C_{20}H_{25}O_6$	0.0
				22	343.1534	$C_{20}H_{23}O_5$	−0.5
				22	303.1227	$C_{17}H_{19}O_5$	0.0
				22	167.0699	$C_9H_{11}O_3$	−0.3
				22	149.0596	$C_9H_9O_2$	−0.1
				22	59.0125	$C_2H_3O_2$	−0.2
M5	421.1855	$C_{22}H_{29}O_8$	−0.2	20	361.1647	$C_{20}H_{25}O_6$	0.1
				20	343.1540	$C_{20}H_{23}O_5$	0.0
				20	167.0701	$C_9H_{11}O_3$	−0.1
				20	149.0958	$C_{10}H_{13}O$	−0.2
				20	59.0125	$C_2H_3O_2$	−0.2
M6	421.1855	$C_{22}H_{29}O_8$	−0.1	20	361.1646	$C_{20}H_{25}O_6$	0.0
				20	343.1541	$C_{20}H_{23}O_5$	0.0
				20	235.0966	$C_{13}H_{15}O_4$	0.1

				20	205.0858	$C_{12}H_{13}O_3$	−0.1
				20	149.0594	$C_9H_9O_2$	−0.2
				20	59.0125	$C_2H_3O_2$	−0.2
M7	421.1855	$C_{22}H_{29}O_8$	−0.1	22	361.1646	$C_{20}H_{25}O_6$	0.0
				22	343.1541	$C_{20}H_{23}O_5$	0.1
				22	235.0966	$C_{13}H_{15}O_4$	0.1
				22	149.0594	$C_9H_9O_2$	−0.3
				22	59.0125	$C_2H_3O_2$	−0.2
M8	421.1855	$C_{22}H_{29}O_8$	−0.1	20	361.1646	$C_{20}H_{25}O_6$	0.0
				20	343.1540	$C_{20}H_{23}O_5$	0.0
				20	235.0965	$C_{13}H_{15}O_4$	0.0
				20	167.0700	$C_9H_{11}O_3$	−0.2
				20	59.0125	$C_2H_3O_2$	−0.2
M9	421.1852	$C_{22}H_{29}O_8$	−0.4	28	361.1645	$C_{20}H_{25}O_6$	0.0
				28	343.1535	$C_{20}H_{23}O_5$	−0.4
				28	167.0700	$C_9H_{11}O_3$	−0.2
				28	149.0594	$C_9H_9O_2$	−0.3
				28	59.0125	$C_2H_3O_2$	-0.2
M10	421.1855	$C_{22}H_{29}O_8$	−0.2	18	361.1646	$C_{20}H_{25}O_6$	0.0
				18	343.1539	$C_{20}H_{23}O_5$	0.0
				18	317.1747	$C_{19}H_{25}O_4$	0.0
				18	167.0700	$C_9H_{11}O_3$	−0.2
				18	59.0125	$C_2H_3O_2$	−0.2
M11	423.2011	$C_{22}H_{31}O_8$	−0.2	20	363.1803	$C_{20}H_{27}O_6$	0.1
				20	345.1696	$C_{20}H_{25}O_5$	0.0
				20	301.1798	$C_{19}H_{25}O_3$	0.0
				20	151.0750	$C_9H_{11}O_2$	−0.3
				20	149.0959	$C_{10}H_{13}O$	−0.2
				20	59.0125	$C_2H_3O_2$	−0.2
M12	423.2010	$C_{22}H_{31}O_8$	−0.3	20	363.1802	$C_{20}H_{27}O_6$	0.0
				20	345.1694	$C_{20}H_{25}O_5$	−0.2
				20	315.1589	$C_{19}H_{23}O_4$	−0.1
				20	169.0857	$C_9H_{13}O_3$	−0.2
				20	149.0958	$C_{10}H_{13}O$	−0.3
				20	59.0125	$C_2H_3O_2$	−0.2
M13	423.2010	$C_{22}H_{31}O_8$	−0.2	28	363.1803	$C_{20}H_{27}O_6$	0.1
				28	345.1697	$C_{20}H_{25}O_5$	0.0
				28	301.1799	$C_{19}H_{25}O_3$	0.0
				28	169.0857	$C_9H_{13}O_3$	−0.1
				28	151.0751	$C_9H_{11}O_2$	−0.2
				28	149.0958	$C_{10}H_{13}O$	−0.2
				28	59.0125	$C_2H_3O_2$	−0.2
M14	439.1958	$C_{22}H_{31}O_9$	−0.4	25	379.1750	$C_{20}H_{27}O_7$	−0.1
				25	361.1645	$C_{20}H_{25}O_6$	0.0
				25	317.1745	$C_{19}H_{25}O_4$	−0.2
				25	169.0857	$C_9H_{13}O_3$	−0.2
				25	165.0908	$C_{10}H_{13}O_2$	−0.2
				25	151.0751	$C_9H_{11}O_2$	−0.2
				25	59.0125	$C_2H_3O_2$	−0.2
M15	439.1958	$C_{22}H_{31}O_9$	−0.4	28	379.1750	$C_{20}H_{27}O_7$	0.0
				28	169.0857	$C_9H_{13}O_3$	−0.2
				28	165.0908	$C_{10}H_{13}O_2$	−0.2

				28	151.0751	$C_9H_{11}O_2$	−0.2
				28	59.0125	$C_2H_3O_2$	−0.2
M16	439.1958	$C_{22}H_{31}O_9$	−0.4	28	379.1750	$C_{20}H_{27}O_7$	0.0
				28	185.0807	$C_9H_{13}O_4$	−0.1
				28	167.0701	$C_9H_{11}O_3$	−0.1
				28	149.0958	$C_{10}H_{13}O$	−0.2
				28	59.0125	$C_2H_3O_2$	−0.2
M17	439.1960	$C_{22}H_{31}O_9$	−0.2	30	379.1751	$C_{20}H_{27}O_7$	0.0
				30	361.1646	$C_{20}H_{25}O_6$	0.0
				30	185.0808	$C_9H_{13}O_4$	0.0
				30	167.0701	$C_9H_{11}O_3$	−0.1
				30	149.0959	$C_{10}H_{13}O$	−0.2
				30	59.0125	$C_2H_3O_2$	−0.2
M18	439.1957	$C_{22}H_{31}O_9$	−0.3	30	379.1750	$C_{20}H_{27}O_7$	0.0
				30	185.0806	$C_9H_{13}O_4$	−0.2
				30	167.0700	$C_9H_{11}O_3$	−0.2
				30	149.0957	$C_{10}H_{13}O$	−0.2
				30	59.0125	$C_2H_3O_2$	−0.2
M19	439.1956	$C_{22}H_{31}O_9$	−0.6	33	379.1749	$C_{20}H_{27}O_7$	−0.2
				33	361.1645	$C_{20}H_{25}O_6$	0.0
				33	169.0857	$C_9H_{13}O_3$	−0.2
				33	165.0907	$C_{10}H_{13}O_2$	−0.2
				33	151.0750	$C_9H_{11}O_2$	−0.3
				33	59.0125	$C_2H_3O_2$	−0.2
M20	439.1958	$C_{22}H_{31}O_9$	-0.4	22	379.1751	$C_{20}H_{27}O_7$	0.0
				22	185.0803	$C_9H_{13}O_4$	−0.5
				22	167.0700	$C_9H_{11}O_3$	−0.2
				22	149.0957	$C_{10}H_{13}O$	−0.4
				22	59.0125	$C_2H_3O_2$	−0.2

CE, collision energy; comp., composition; exp., experiment.
